# Molecular evolution of coxsackievirus A24v in Cuba over 23-years, 1986–2009

**DOI:** 10.1038/s41598-020-70436-w

**Published:** 2020-08-13

**Authors:** Magilé C. Fonseca, Mario Pupo-Meriño, Luis A. García-González, Sonia Resik, Lai Heng Hung, Mayra Muné, Hermis Rodríguez, Luis Morier, Heléne Norder, Luis Sarmiento

**Affiliations:** 1 Virology Department, Center for Research, Diagnosis and Reference, Institute of Tropical Medicine “Pedro Kourí” (IPK), Novia del Mediodía Km 61/2, La Lisa, Marianao 13, P.O. Box: 601, Havana, Cuba; 2 Departamento de Bioinformática, Centro de Matemática Computacional, Universidad de las Ciencias Informáticas (UCI), Havana, Cuba; 3 Departamento de Ciencias de la Computación, Centro de Investigación Científica y de Educación Superior de Ensenada, Ensenada, Baja California México; 4 Cell Culture Laboratory, Center for Research, Diagnosis and Reference, Institute of Tropical Medicine “Pedro Kourí” (IPK), Havana, Cuba; 5 Department of Microbiology and Virology, Biology Faculty, Havana University, Havana, Cuba; 6 Department of Infectious Diseases/Virology, Institute of Biomedicine, Sahlgrenska Academy, University of Gothenburg, Gothenburg, Sweden; 7 Immunovirology Unit, Department of Clinical Sciences, Skåne University Hospital, Lund University, Malmö, Sweden

**Keywords:** Viral infection, Phylogenetics

## Abstract

Coxsackievirus A24 variant (CVA24v) is a major causative agent of acute hemorrhagic conjunctivitis outbreaks worldwide, yet the evolutionary and transmission dynamics of the virus remain unclear. To address this, we analyzed and compared the 3C and partial VP1 gene regions of CVA24v isolates obtained from five outbreaks in Cuba between 1986 and 2009 and strains isolated worldwide. Here we show that Cuban strains were homologous to those isolated in Africa, the Americas and Asia during the same time period. Two genotypes of CVA24v (GIII and GIV) were repeatedly introduced into Cuba and they arose about two years before the epidemic was detected. The two genotypes co-evolved with a population size that is stable over time. However, nucleotide substitution rates peaked during pandemics with 4.39 × 10^−3^ and 5.80 × 10^−3^ substitutions per site per year for the 3C and VP1 region, respectively. The phylogeographic analysis identified 25 and 19 viral transmission routes based on 3C and VP1 regions, respectively. Pandemic viruses usually originated in Asia, and both China and Brazil were the major hub for the global dispersal of the virus. Together, these data provide novel insight into the epidemiological dynamics of this virus and possibly other pandemic viruses.

## Introduction

Human enteroviruses are small, non-enveloped viruses belonging to the *Enterovirus* genus of the *Picornaviridae* family^1^. The genome is a single-stranded positive sense RNA molecule of approximately 7.4 kb and possesses a long open reading frame (ORF) that is flanked on both ends by the 5′ and 3′ untranslated regions. The ORF encodes a polyprotein, which is cleaved to form seven non-structural proteins (2A, 2B, 2C, 3A, 3B, 3C, and 3D) and four structural proteins (VP1, VP2, VP3, and VP4). Human enteroviruses comprise four species, namely, species *Enterovirus A–Enterovirus D*. Each of these four species is formed by five to 63 different types, with no cross neutralization, i.e. infection with one type does not infer immunity against another type^1^. Recombination has often been reported either between members of the same or different human enteroviruses species. The recombination “hotspot” regions are located not only between the structural and non-structural coding regions but also between the 5′ non-coding region and the protein-encoding region of human enteroviruses^2^.

Coxsackievirus A24v (CVA24v), an antigenic variant of the CVA24 strain (member of species *Enterovirus C*), was first isolated from an outbreak of acute hemorrhagic conjunctivitis (AHC) in Singapore in 1970. Afterwards, CVA24v has been identified as the major causative agent of AHC outbreaks worldwide^3–5^. Currently, four genotypes of CVA24v have been described (I–IV), which have been responsible for major global epidemics of AHC^5–7^. Several studies have investigated the genetic diversity and molecular evolution of CVA24v strains during periods varying from 4 to 20 years^5–7^. Most of these studies were performed on strains of CVA24v that originated from Asia, however, there have been few phylogenetic studies of CVA24v-related AHC outbreaks in the Americas, as in Brazil, French Guiana and Mexico^8–11^.

The first description of AHC in Cuba is related to an outbreak caused by enterovirus 70 (member of species *Enterovirus D*) in 1981, with more than 800,000 cases^12,13^. From then until 2009, five major outbreaks of AHC due to CVA24v have been documented by the Cuba’s National Epidemiologic Surveillance. The first CVA24v AHC outbreak emerged in the municipality of Isla de la Juventud in September 1986 and rapidly spread across the country causing an epidemic of 607,159 cases during the 1986–1987 period. The second nationwide outbreak occurred in 1992–1993 with 90,884 cases^12,13^. It is still unclear how the virus emerged in 1993 because there is no published data on possible AHC outbreaks in the Caribbean islands during this period. Only one sequence of CVA24v isolated in the Dominican Republic during 1993 is available in GenBank. In addition, there were only few reported AHC outbreaks around the world during the early 1990s, those reported occurred in Asia and Africa^14–16^.

The third Cuban AHC outbreak occurred in 1997 spring with 137,136 cases. It began in Havana city and rapidly spread to the entire country^12,13^. During 1997 and 1998, there were numerous outbreaks caused by CVA24v in several Latin American countries, as Antigua/Barbuda, Bahamas, British Virgin Islands, St. Christopher/Nevis, Trinidad and Tobago, Suriname, Puerto Rico and Mexico^17,18^. CVA24v epidemics were also reported in China and India in 1997 and 1999, respectively^19,20^. The fourth large-scale outbreak of AHC (171,910 cases) due to CAV24v in Cuba occurred from July through December 2003^12^. The fifth Cuban outbreak of AHC (72,138 cases) occurred in 2008–2009^21^. There were reports of AHC epidemic in Honduras, Brazil, Taiwan, China and India between 2007 and 2010^8,22–25^ .

Given the fact that CVA24v outbreaks has been occurring regularly in Cuba^12^, this study was performed to form basis for the understanding of the evolutionary and epidemiological dynamics of CVA24v in the Americas. The sequences of CVA24v strains from five AHC epidemics in Cuba were used to determine the viral phylodynamics and the phylogenetic relationships between strains involved in the outbreaks and strains isolated from other parts of the world during the same periods.

## Results

### Identity analysis

A total of 157 partial 3C sequences (507 nt) and 149 partial VP1 sequences (234 nt) were obtained from 159 strains of CVA24v isolated during the epidemic periods from 1985 through 2005 (Supplementary Table S1). Our failure to obtain sequence CVA24v products in 6.3% (VP1) and 1.3% (3C) of CVA24v isolates could be attributed to the degradation of viral RNA due to prolonged storage or multiple freeze*–*thaw cycles. In addition, failure to achieve amplification in VP1 region to a greater extent than 3C may be due to VP1 primers' reliance on conserved amino acid motifs specific to the *Enterovirus* genus. The 3C and VP1 sequences of the Cuban strains had more than 88.3% nucleotide and 93.0% amino acids identity with the corresponding regions of the prototype EH24_70_Singapore 1970 (Supplementary Table S2). The Cuban CVA24v strains isolated during two consecutive years in four outbreaks of AHC (i.e., 1986–1987, 1992–1993, 2003/2005, 2008–2009) were greater than 97.0% identical at the nucleotide level during each outbreak (Supplementary Table S3).

### Molecular epidemiology of CVA24v in Cuba

The removal of the identical sequences in 3C and VP1 region in a random manner using the ElimDupes tool (https://www.hiv.lanl.gov) resulted in 54 Cuban 3C sequences that were compared with 83 partial 3C sequences from strains isolated in 17 countries. Likewise, 35 Cuban VP1 sequences were compared with 95 published sequences of CVA24v strains isolated from 18 countries. Sequences of Cuban strains and globally isolated strains that were obtained from the GenBank database are listed in Supplementary Table S4 and S5.

No saturation was observed neither in the plot of the absolute number of transitions and transversions versus genetic distance nor in the Xia test (Supplementary Fig. S1). The noise analysis showed a good resolution of quartet trees with only 8.4% and 16.5% of points located into partly solved and unresolved quartet area for 3C and VP1, respectively (Supplementary Fig. S2). In addition, no recombination events in the 3C or VP1 regions were demonstrated within each CVA24v sequence selected or between CVA24v and other *Enterovirus C* strains (Supplementary Table S6 and S7).

**Figure 1:**
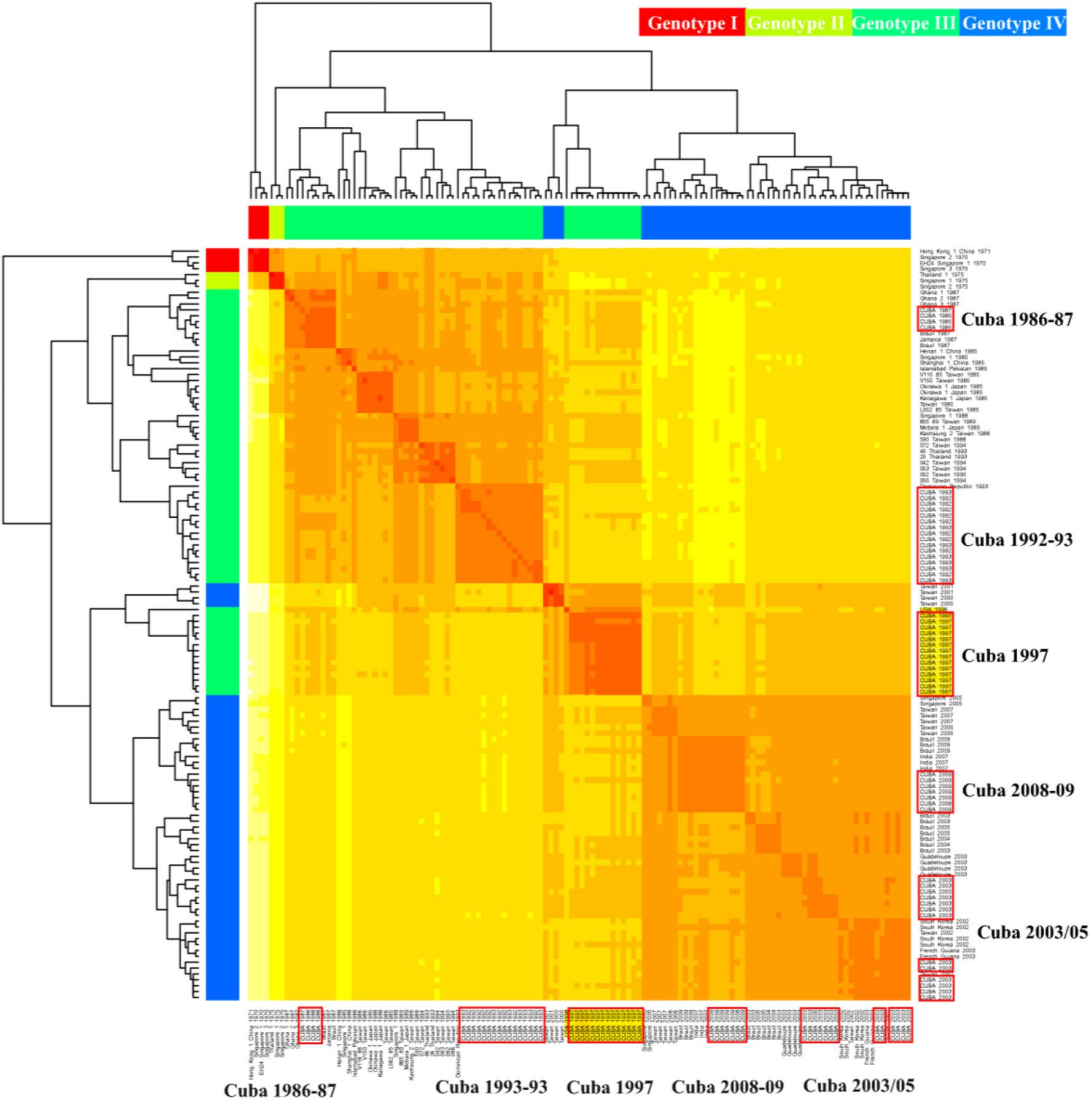
Heat map from nucleotide identity matrix of the 3C region alignment. Sequences were classified by GI-IV Genotypes described by Chu et.al.^7^. Genotypes are indicated by the color legend on the top and in the left side in correspondence with the genotype’s clade distribution. Cuban (1997) and USA (1998) sequences are highlighted in yellow. Cuban sequences from five AHC epidemics are highlighted in red rectangles.

The GTR nucleotide substitution model with a gamma rate distribution plus invariable sites (GTR + G + I) was identified as the best-fit evolutionary model by jModelTest v2.1.4 program^26^. Heat maps of average nucleotide identity matrix revealed that CVA24v strains isolated from the Cuban AHC epidemics in 1986–1987 and 1992–1993 belonged to genotype III. While this may be expected, given the time periods they were isolated, it is noteworthy that Cuban CVA24v strains isolated in 1997 were clustered into genotype IV. Remarkably, a notably higher intragroup identity than intergroup identity was demonstrated in both coding regions. This suggests that the accepted classification of CVA24v genotypes should be reconsidered (Fig. 1 and Supplementary Fig. S3).

**Figure 2:**
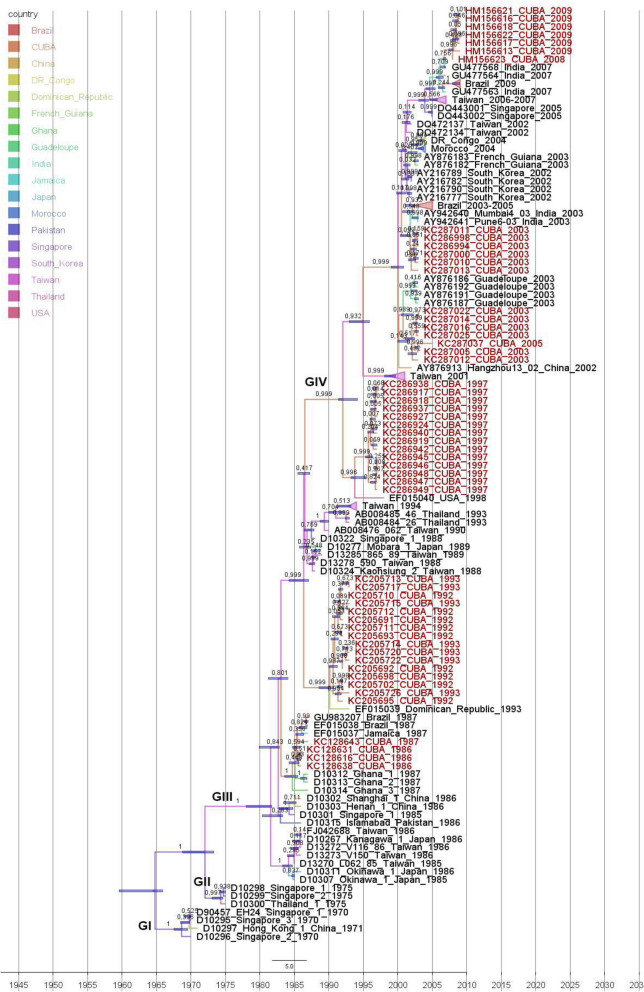
Maximum clade credibility (MCC) phylogeny tree of 3C sequences (507 nt) of Cuban (n = 54, in red) and worldwide CVA24v strains (n = 83). For each branch, the color indicates the most probable location state of their descendent nodes. Bars at nodes indicate 95% HPDs of TMRCAs. Branches forming genotypes GI-GIV are shown. The sequences are indicated by GenBank accession number, strain name, country and year of isolation.

**Figure 3:**
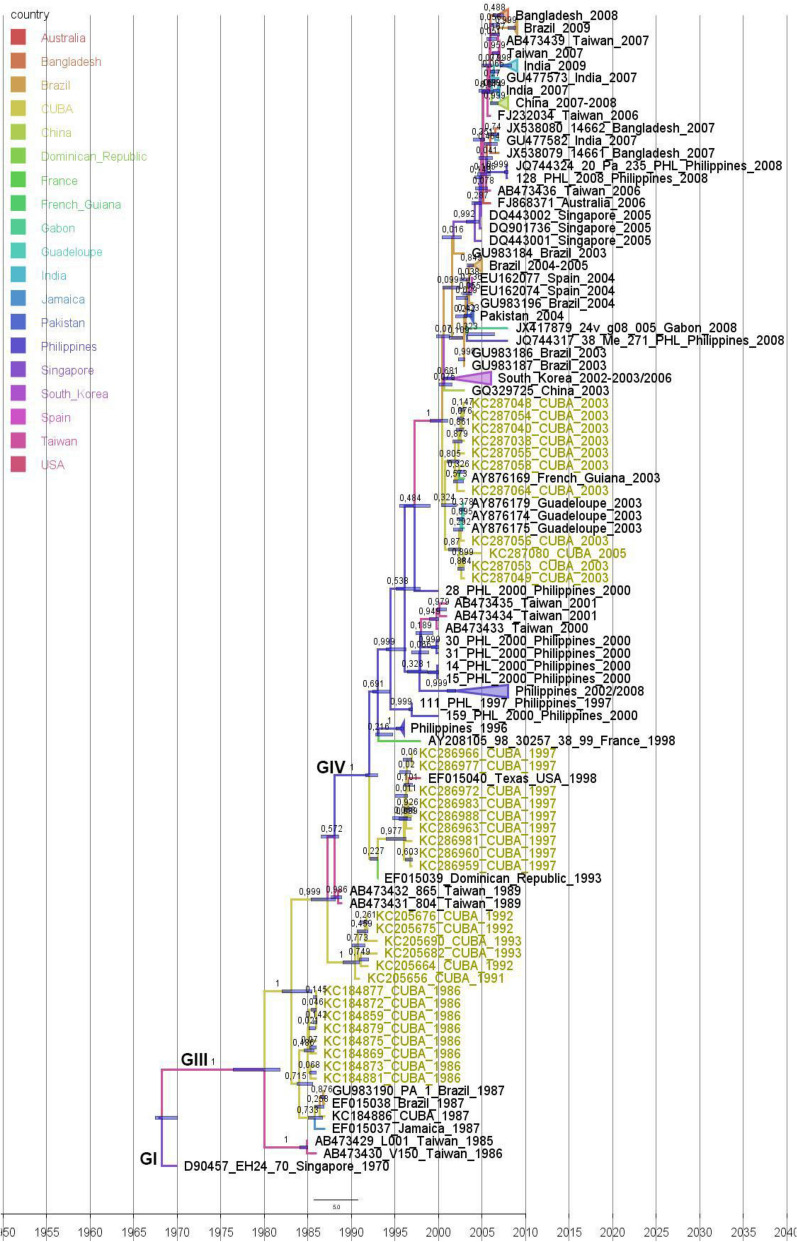
Maximum clade credibility (MCC) phylogeny of VP1 sequences (234 nt) of Cuban (n = 35 in yellow) and worldwide CVA24v strains (n = 95). For each branch, the color indicates the most probable location state of their descendent nodes Bars at nodes indicate 95% HPDs of TMRCAs. Branches forming genotypes GI-GIV are shown. The sequences are indicated by GenBank accession number, strain name, country and year of isolation.

The phylogenetic trees based on the 3C sequences showed that the Cuban CVA24v strains isolated in 1986 and one strain isolated in 1987 formed a clade with CVA24v strains isolated during outbreaks in Jamaica, Brazil and Ghana in 1987 (> 98% nucleotide identity) (Fig. 2). These results confirm previous epidemiological data suggesting that 1986 outbreak of AHC in Cuba originated from the introduction of CVA24v by Ghanaian students that arrived to Isla de la Juventud during the summer of 1986^13^. Phylogenetic analysis based on VP1 region revealed a close relation between Cuban strains and Latin-American strains isolated in 1987 (98.7–99.1% nucleotide identity) (Fig. 3). The VP1 region of the Ghanaian strains was not available in GenBank.

Sixteen Cuban CVA24v strains isolated during the 1992–1993 AHC outbreak formed a clade with a Dominican strain isolated in 1993, sharing 97.8–99.8% nucleotide identity in 3C region (Fig. 2). In the VP1 region, the six Cuban strains from 1992 to 1993 formed an independent clade, separated in time to the common ancestor of the Dominican strain. Curiously, the Dominican strain was included within the clade formed by the Cuban 1997 sequences (Fig. 3).

The phylogenetic trees based on the VP1 and 3C sequences of Cuban 1997 CVA24v strains showed that these strains formed a separate clade together with a strain isolated in the Unites States in 1998 (Figs. 2, 3).

The inconsistence in the 1990s topology suggests the occurrence of at least one intratypic recombination event, which was verified by testing complete sequences of some CVA24v and other *Enterovirus C* strains (Supplementary Table S8) using additional recombination methods included in the RDP4 program^27^. This analysis showed that the Dominican strain isolated in 1993 is a recombinant virus with a major parental sequences (JN228097: Human coxsackievirus A24 variant South Korea 2004 and EF015037: JAM87-10628) that share similarity with Cuban strains isolated the same year or contemporary, which were classified within genotype III and IV (Supplementary Table S9).

Beyond inconsistence between 3C and VP1 trees topologies, the sequences from the late 1990s and early 2000s are located into the same branch forming a single and well-defined clade. This observation in conjunction with the fact that heat map analysis displays a close nucleotide identity between sequences dating from the late-1990s and the early-2000s*,* support the idea of considering late 1990s sequences as the first occurrence of Genotype IV.

Phylogenetic analysis of the 3C region of 12 Cuban strains isolated in 2003 and one strain in 2005 revealed a separation of the strains into two clades (Fig. 2). One of the clades was formed by six strains from 2003 and the strain from 2005 together with four isolates from an AHC outbreak in Guadeloupe in 2003, sharing 95.6–99.8% nucleotide identity. The other clade was formed by six Cuban strains from the 2003 AHC outbreak in Cuba, two Indian strains isolated in 2003 and CVA24v strains that had caused the Brazilian AHC outbreaks during 2003–2005 (97.4–99.6% nucleotide identity). The analyses of the VP1 region revealed that Cuban strains also formed two clades (Fig. 3). In the first clade, three strains from 2003 and one strain from 2005 clustered with three strains isolated in Guadeloupe in 2003, which were 98.2–99.1% identical at nucleotide level with the Cuban strains. In the other clade, seven of the Cuban strains from 2003 clustered with a strain isolated from an outbreak in French Guiana in the same year, sharing 98.2–100% nucleotide identity. Analysis of 3C gene sequences of Cuban CVA24v strains obtained during the 2008–2009 AHC outbreak^21^ showed a clade together with strains isolated from India in 2007 and during the AHC outbreak of 2009 in Brazil, sharing 98.2–99.8% nucleotide identity (Fig. 2).

### Phylodynamic analysis of CVA24v based on the sequenced 3C and VP1 regions

Topologies based on the sequences of the 3C and VP1 regions were similar to each other (Figs. 2, 3). The analysis revealed a monophyletic origin of all strains that diverged from those isolates that circulated during the first epidemic in Singapore in 1970^5,28^. The CVA24v topologies exhibits an unbalanced tree structure towards a “ladder-like” shape, characterized by a sequential replacement of the existing viral populations by other emergent ones on a global scale, and a limited genetic diversity observed through time. Additionally, short terminal branches with low support values were observed, which may suggests that the grouped sequences can be explained by different topology^5,29–31^.

**Figure 4:**
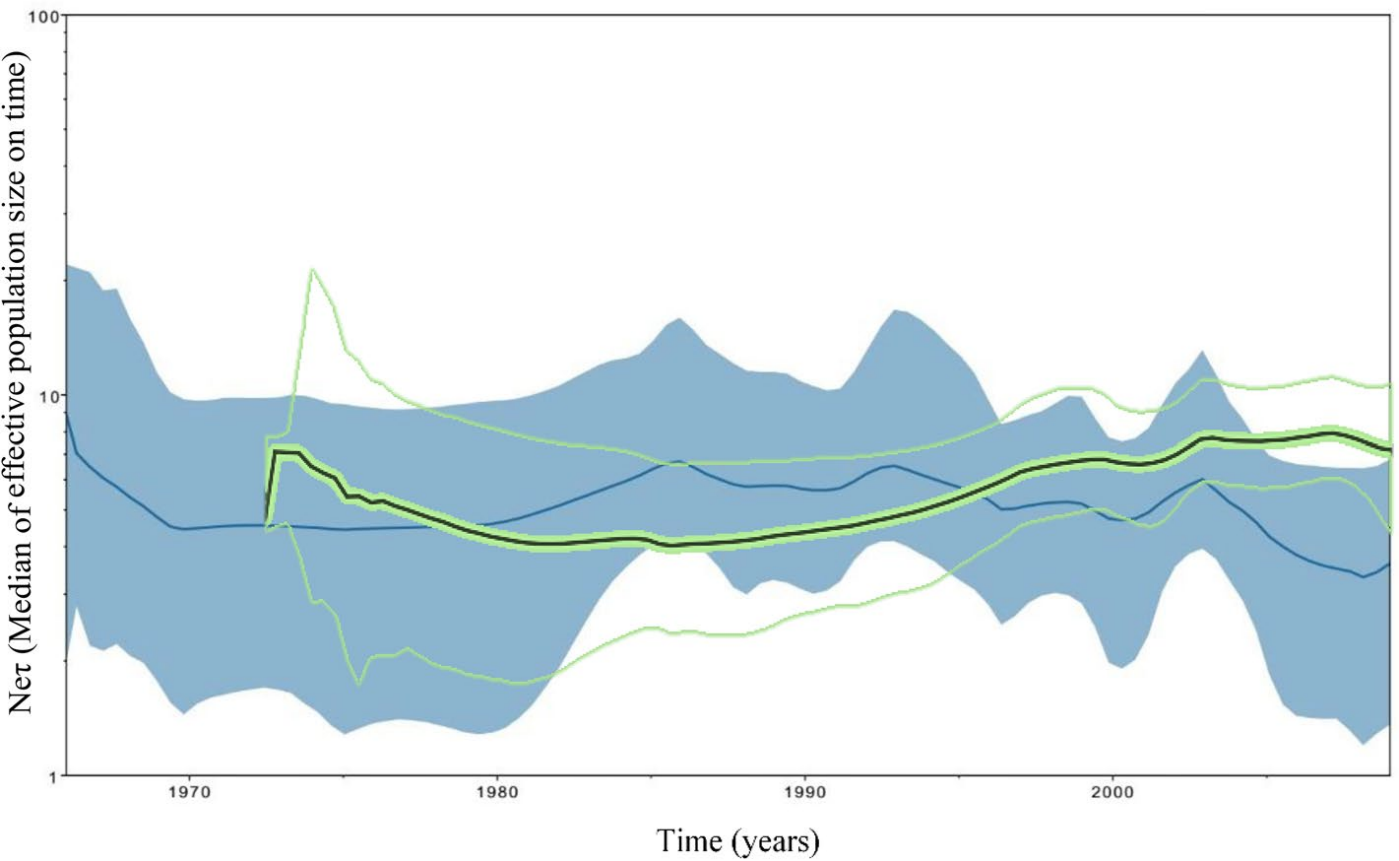
Bayesian skyride plot of Cuban and world-wide of 3C (137 sequences, 507 nt) coding region (blue) and VP1 (130 sequences, 234nt) coding region (green) of CVA24v. The x-axis is the time scale (years) and the y-axis is the logarithmic Neτ scale (Ne is the median of effective population size and τ is the generation time). The thick solid line indicates the median estimates and the shaded area indicates the 95% HPD.

Skyride plots were used for reconstructing temporal variations in the genetic diversity of 137 Cuban and world-wide CVA24v strains for 3C coding region as 130 for VP1 coding region (Fig. 4). The analysis showed that the effective population size of the virus is stable over time with slight increases, coinciding with the pandemic periods within the four epidemiological periods of CVA24v^5,7^. This was confirmed when both the 3C and the VP1 regions were analyzed (Fig. 4). The population was emerging by the 1970s when the virus appeared with an effective median population size of 9.52 for VP1 and 7.04 for 3C. Thereafter, it decreased to 4.43 for 3C and to 2.79 for VP1, coinciding with the limited CVA24v circulation in the Southeast Asia and Indian regions prior to 1985. A virulent period occurred after the mid-1980s and was characterized by many outbreaks worldwide. This was represented by a Neτ increase for 3C (Neτ = 6.70) and a weak increase in VP1 (Neτ = 2.95–2.99) between 1984 and 1988. Thereafter a silent period occurred during the 1990s with few outbreaks globally, although two peaks in the 3C region (Neτ = 6.53 and Neτ = 5.24) were obtained as well as a slight increase in the VP1 region between 1997 and 1999 (Neτ = 5.94–6.52; Fig. 4). Importantly, these peaks were in correspondence with the two epidemic periods at the beginning of the 1990s and after 1996^14–17^. Finally, the viral population size increased exponentially between 2002 and 2003 (3C Neτ = 6.01 and VP1 Neτ = 8.1). Furthermore, other peak for VP1 (Neτ = 8.46) was demonstrated between 2007 and 2008, which is consistent with the re-emerging period in the 2000s where the virus rapidly spread to many countries throughout the world^5,7,24^.

The confidence intervals of the skyline plots are so wide that they do not show significant increases or decreases during particular periods. Thus, effective population size is stable over time when both the 3C and the VP1 partial regions were analysed (Fig. 4). The greatest uncertainty is observed in the early 1970s period when the virus appeared and population is supposed to be emerging. The insufficient sampling due to the small number of sequenced strains in this period could bring us to misestimate the effective population size, as population dynamics is mostly reconstructed as a result of the simulation instead of being data-driven. Those confidence intervals become narrower in subsequent periods, but still there is a high variability which is most notably in 3C region. No artefacts associated with the convergence of the MCMC runs were detected, so insufficient sampling or the low variability of the virus could have the major role in the uncertainty on estimations.

Based on divergence time analyses, the time for the most recent common ancestor (95% HPD) for the CVA24v strains was estimated around the 1960s (Table 1). The evolution into a new genotype (I–IV) likely emerged between 4 and 5 years before they spread globally, and some caused pandemics. The analyses also indicate that some CVA24v strains may have been present in Cuba for 1 to 2 years prior to the large outbreak (Table 2).

**Table 1:** The TMRCAs estimated for CVA24v genotypes (I-IV) described by Chu et al., based on Bayesian sequence analysis of partial 3C and VP1 regions.

CV-A24v genotypes	TMRCAs (95% HPD)/(N )	
	3C	VP1
GI	1964.8 (1959.5–1965.9)/(4)	1968.1 (1967.5–1970)/(1)
GII	1972 (1968.8–1973.3)/(3)	NA
GIII	1981.7 (1978–1981.7)/(65)	1980 (1976.4–1981.9)/(23)
GIV	1995 (1993–1995.9)/(65)	1996 (1995.1–1997.8) /(106)

N, number of sequences; NA, VP1 sequences not available at Genbank.

**Table 2:** The TMRCAs estimated for Cuban CHA epidemics based on 3C and VP1 coding regions of CVA24v strains.

Cuban AHC epidemics	TMRCAs (95% HPD)/(N)	
	3C	VP1
1986–1987	1985.3 (1984.2–1985.6)/(4)	1984 (1983.8–1985.6)/(9)
1992–1993	1990.6 (1990–1991.2)/(16)	1990.4 (1989–1990.9)/(6)
1997	1995.7 (1995.3–1996.4)/(14)	1996 (1994–1996.2)/(9)
2003/2005^a^	2002 (2001.1–2002.4)/(6)	2002.3 (2001.2–2002.5)/(7)
	2001.9 (2001.5–2002.6)/(7)	2002.8 (2001–2002.4)/(4)
2008–2009	2007.9 (2007.4–2008)/(7)	ND

N, number of sequences; ND, non determined, the VP1 region was not sequenced.

^a^The TMRCAs for each clade of Cuban sequences obtained.

The mean substitution rate was estimated to be 4.39 × 10^−3^ nucleotide substitutions per site per year (s/s/y) (95% HPD 3.48 × 10^−3^–5.36 × 10^−3^) for 3C (137 sequences) and 5.80 × 10^−3^ (95% HPD 4.37 × 10^−3^–7.32 × 10^−3^) for VP1 (130 sequences).

The discrete phylogeographic analysis revealed 25 transmission routes for VP1 and 19 routes for 3C genomic region (Table 3 and Supplementary Fig. S4). The analyses showed that the strains causing outbreaks in Cuba usually originate in Asia, and that both China and Brazil play key roles in reseeding the virus globally (Table 3 and Supplementary Fig. S4).

**Table 3:**
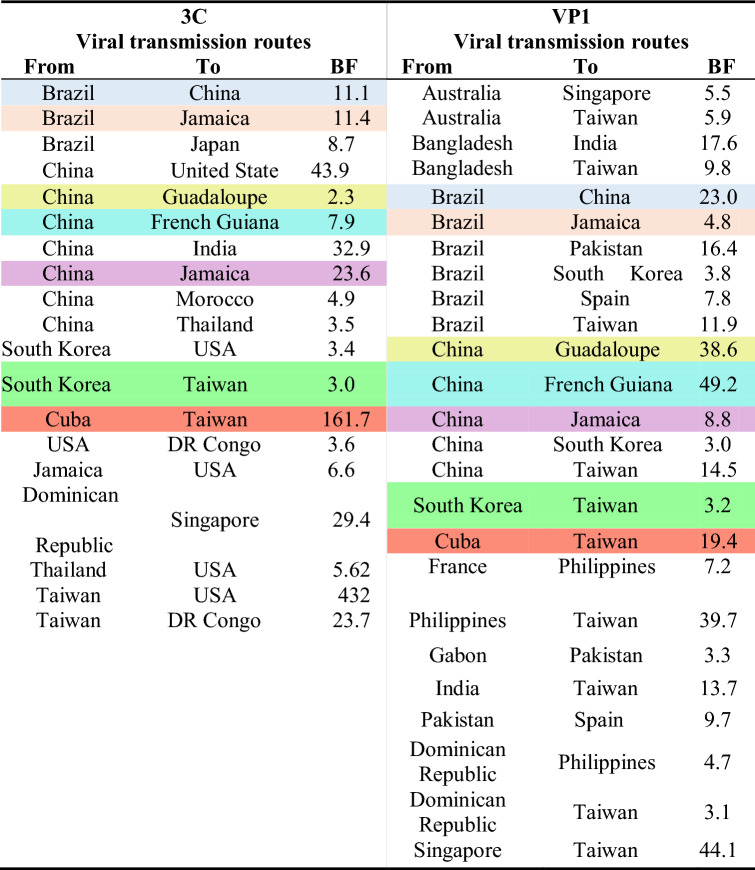
Epidemiologic connections routes of CVA24v with BF > 3.0 based on 3C and VP1 genomic regions.

Highlighted with the same color the common routes obtained in both analyses.

A reconstructed network analysis based on a random walk based community detection algorithm (Walktrap analysis^32^) was performed to identify molecular signatures for the spreading of CVA24v strains. In agreement with the results obtained in the phylogeographic analysis, strains isolated in Cuba were shown to be involved in one of two major networks, which included strains from Asia (Supplementary Fig. S5).

Both analyses identified four different global transmission routes of CVA24v to Cuba: (1) the route from China to Jamaica for the 1986–1987 Cuban AHC epidemics; (2) four routes from China, South Korea, Thailand and Taiwan to USA, which were obtained when analysing the 3C region of strains isolated during the 1997 AHC outbreak; (3) two routes from China to French Guiana and from China to Guadeloupe for the 2003 outbreak; and (4) a route from China to India for the 2008–2009 Cuban AHC outbreaks (Table 3 and Supplementary Fig. S5).

## Discussion

Cuba has been an active area of CVA24v circulation in the Americas since the mid-1980s*.* This study identified that each epidemic of AHC in Cuba was caused by a genetically distinct CVA24v strain, and that the epidemics strains had emerged a few years before they gave rise to new epidemics. We also demonstrated a sequential development of the CVA24v genotypes, each involved in pandemics, similar to what has been shown for other viruses causing major outbreaks or global spread such as influenza A virus, HIV and EV-D68^33,34^. Our finding suggests that the CVA24v strains that caused epidemics of AHC in Cuba and the Americas region came from Asia, which is consistent with previous studies on epidemiological data recorded during AHC outbreaks^8,35–38^. Given the close genetic relationship between the Cuban strains and those from common destination countries in Latin America, it is likely that the epidemic strains may have reached some countries in the region before it caused epidemic in Cuba. The strains could have been brought into Cuba by the frequent travelling between Cuba and Latin American countries. Taken together*,* these results provide insight into the epidemiological dynamics of CVA24v and possibly other pandemic viruses.

There has been a great deal of controversy over the last years regarding which region of the genome is more informative from the phylogenetic point of view^7,39,40^. The first phylogenetic studies of the CVA24v genomes were based on the non-structural 3C region, however, recently analysis of this genomic region has been replaced by the analysis of the structural VP1 region^5,6,14,39^. In this work, both regions were shown equally informative for phylogenetic and phylodynamic analyses. Notably, phylogenetic analyses showed similar outcomes for the two genomic regions, except for the Dominican Republic strain isolated in 1993. This data indicates the occurrence of few recombination events on CVA24v within VP4 structural protein and within 2B nonstructural protein, which is in sharp contrast to what has been repeatedly reported for other *Enterovirus C* strains^41^.

The lack of interspecies recombination events for CVA24v is in agreement with previous reports, suggesting a genomic barrier for recombination when complete 3C and VP1 of CVA24 were analysed^28,40^. However, intratypic recombination events in CVA24v may occur in nonstructural rather than structural proteins as shown for most enteroviruses^40,42^. Clearly, further studies are needed to elucidate possible influence of these recombinations on the development of pandemic CVA24v strains.

The fact that CVA24v has been present in Cuba for 1–2 years preceding the epidemic peak raise the possibility that the virus may has circulated undetected in Cuba before the epidemic was detected through epidemiological and laboratory surveillance. We envisage three possible scenarios to explain the viral circulation during inter epidemics periods, (1) CVA24v circulate at low levels and escape the surveillance system since asymptomatic infection is the most common outcome, (2) the virus cause symptoms that are not commonly associated with CVA24*-*related outbreak of AHC^15,43^ and (3) the virus can persist in a human population due to its ability to nearly escape the humoral immune response. In support of this, recurrent AHC epidemics have been associated with a decline in neutralizing antibody titers against CVA24v after epidemics^44–47^. Indeed, this has been observed in the Cuban population during the 1986 and 1997 AHC outbreaks^48,49^. Thus, the evolution of the persistent strains needs to be investigated to increase our understanding of low-level transmission of the strains. Further work is necessary to determine if there are several strains circulating when a new epidemic occurs or if the previous strain will be completely eradicated due to the occurrence of a new more virulent CVA24v strain. This knowledge will constitute a basis for best management of future AHC outbreaks.

The time between the appearances of the most common ancestors for the four CVA24v genotypes confirms the high epidemic potential of this virus since new variants evolve fast and become epidemic or pandemic variants. The analysis of the CVA24v strains revealed a sequential development of the genotypes, with strains belonging to genotype IV isolated during the AHC outbreak in Cuba in 1997. These strains were similar to strains isolated in 1996 in the Philippines and in France and the United State in 1998, which indicate an earlier occurrence of genotype IV during mid-1990s, than in the beginning of 2000s^7^. Other studies on CVA24v sequences have reported similar topology of the phylogenetic trees as shown in this study, even if these studies did not relate to time of isolation of the strains or ancestral relationships between the sequences^5,10,25,37,38^. Genotypes of CVA24v have been described; however, the definition of a subgenotype is more obscure. Several studies have shown spatial–temporal difference between strains from different countries by using the 3C and VP1 coding regions or complete genome^6,23,38,40,50,51^. Many of these studies have used different definitions of the strains analysed. Therefore, a consensus is necessary in order to obtain a better classification of CVA24v regarding to which genomic region to analyse and which reference sequences can be chosen by periods and geographical origin.

Repeatedly worldwide spreads of CVA24v mainly from Asia to the rest of the world was shown by the analysis of the viral sequences in this study. This may indicate that there is a higher potential for CVA24v variants to evolve into strains with pandemic potential on the Asian continent, where there is a high density of susceptible hosts. The pandemic strains may also have evolved from previous pandemic strains by accumulating considerable genetic changes during its global transmission. In this scenario, new virus variants that escape from human immune surveillance against the prior pandemic virus can emerge when it is re-introduced into Asia causing new outbreaks and pandemics. This circle with global viral transmissions may take 3 to 7 years as shown previously^14–17,19^. Finally, the re-emerging period during the 2000s has been characterized by extremely rapid spread of the virus throughout the world and was represented by exponential growth of the population size in 2002–2003 and 2007–2008. This is consistent with the epidemiological data from the two pandemic periods observed from the early 2000s to 2004 and around 2005^5,7,24^. Even though such behaviour was not supported in the present study in what respect to population size growth, it did in what concerns rapid spread of the virus throughout the world. Further immunological and molecular studies are needed to elucidate mechanisms and factors determining the re-emergence of CVA24v pandemic strains.

This study has some limitations with regard to, (1) the sanger methodology used for sequencing; which might have resulted in an underestimation of the genetic diversity of the CVA24v strains (2) the analysis was performed only on partial and not complete genomes; (3) the few availability of the CVA24v sequences from all affected regions as from all the pandemics periods to get a better understanding of the evolution and dissemination of this virus; (4) the intrinsic limitations of the phylogenetic analysis and software*.* Despite these limitations, this study is one of the largest studies performed in the Americas regarding the origin, evolution and routes of transmission of CVA24v in the region. It also includes sequences of CVA24v strains isolated between 1986 and 2009 from both the eastern and western hemispheres. Overall, our findings resolve a long-standing question of when and where the epidemics CVA24v could be originated in Cuba. The unexpected detection of strains of CVA24v belonging to genotype IV during mid-1990s highlights the need to revisit outdated origin of genotype IV. Moreover, they underscore the importance of understanding the global evolution of CVA24 and their pandemic threat. The results of the study not only shed light on the genetic diversity, evolution and global transmission of CVA24v but may also help in identifying new control strategies for future epidemics at the national, regional and global level.

## Methods

### Viruses

Archived CVA24v strains (n = 159) used in this study were obtained from National Reference Laboratory for Enteroviruses at the Pedro Kourí Tropical Medicine Institute. The strains were isolated from conjunctival swabs (n = 125), feces (n = 32), nasal swab (1) and pharyngeal swab (1) of patients with AHC during the Cuban outbreaks of 1986–1987, 1992–1993, 1997 and 2003–2005 (Table 4). All strains were isolated on Hep2 (HeLa derivative, ECACC 86030501) cells and the identity of the isolate was confirmed by neutralization tests with type-specific antiserum and PCR^48,49,52^.

**Table 4:** Epidemiological data and number of CVA24v strains from Cuban AHC epidemics. *Source*: National Enterovirus Reference Laboratory. Pedro Kourí Institute of Tropical Medicine (IPK).

Cuban AHC epidemics (year)	Number of cases reported	Collection month(s)	Number of stored strains	Sequenced strains	Specimens sources of the strains
1986	596,445	September	27	27	Conjunctival swabs
1987	10,714	August–September	8	8	Conjunctival swabs
1992	3,363	October–December	22	22	Conjunctival swabs
1993	87,807	September	15	15	Conjunctival swabs
1997	137,136	July–September	43	43	Conjunctival swabs (25) Feces (16) Nasal swab (1) Pharyngeal swab (1)
2003	171,910	June–August, November	39	39	Conjunctival swabs (23) Feces (16)
2005	59	November	5	5	Conjunctival swabs
Total			159	159	Conjunctival swabs (125) Feces (32), Nasal swab (1) Pharyngeal swab (1)

### RNA extraction and cDNAs synthesis

Nucleic acids were extracted from 250 µl of infected cell culture supernatant using Trizol (Life Technologies. Gibco BRL; Grand Island, N.Y.USA) and precipitated with isopropanol. The pellet was washed with l ml of 75% ethanol, dried and suspended in 50 µl of diethylpyrocarbonate (DEPC)-treated water. RNA was reverse transcribed to cDNA with Oligo(dT)_20_ using ThermoScript RT-PCR System (Life Technologies. Gibco BRL Inc.) according to the manufacturer’s instructions.

### PCR amplification and sequencing of VP1 and 3C regions

Published primers of the 5′-half of the VP1-coding region of the genome (primer 222 [5′-CICCIGGIGGIAYRWACAT-3′] and primer 224 [5′-GCIATGYTIGGIACICAYRT-3′]) were used for PCR amplification and sequencing^53^. The 3C protease region was amplified with primer pair D1 [5′-TACAAACTGTTTGCTGGGCA-3′] and U2 [5′-TTCTTTTGATGGTCTCAT-3′]^14^. Amplified products were purified using E.Z.N.A Cycle-Pure Kit (Omega Bio-tek. Inc.) according to the manufacturer’s instructions. Cycle sequencing reactions with both primers pair were performed by the ABI BigDye Terminator v 3.1 Cycle Sequencing Kit (Applied Biosystems). The ABI PRISM 3100 Genetic Analyser (Applied Biosystems) was used for electrophoresis and data collection. The sequences data were submitted to GenBank (accession numbers KC128616-KC128648, KC184859-KC184890, KC205656-KC205727, and KC286913-KC287081).

### Identity analysis

Sequences obtained were edited and the percentage similarity between the Cuban strains and prototype strain EH24_70_Singapore 1970 were determined using the Bioedit v7.0.5.3 program^54^.

### Phylogenetic analysis

The analysis of both coding regions were conducted with the following algorithm: alignment (multiple sequences alignment), quality control, evolutionary model selection, testing phylogeny and phylodynamics (modified from^55^). For the phylodynamic step, inference of population parameters and phylogeographic analysis were performed.

### Data set and multiple alignment

Sequences from strains isolated from different geographical regions during years or contemporary when outbreaks had occurred in Cuba were obtained from GenBank. Sixteen 3C gene sequences of CVA24v obtained in our previous studies were also included in the phylogenetic analysis. The sequences were derived from CVA24v strains isolated during the Cuban AHC outbreak in 2008 (n = 6) and 2009 (n = 10)^21^. Duplicate sequences were eliminated using the ElimDupes tool from the Alamos HIV database (https://www.hiv.lanl.gov). Multiple sequence alignments were performed by MAFFT v7 by using L-INS-I protocol^56^.

### Quality control

The quality of the sequence alignments of both coding regions resulted from the filtering of duplex sequences. The nucleotide sequences of partial 3C and VP1 regions of Cuban and world-wide CVA24v strains (Supplementary Table S4 and S5) were explored using the following tests. Saturation effects were investigated by plotting the absolute number of transitions and transversions versus genetic distance for all CVA24v selected, using the DAMBE v6.0 software^57^. Additionally, the standard statistical test of Xia et al.^58^ was performed to assess whether a set of molecular sequences had experienced substitution saturation. Genetic distances were calculated with the general time reversible (GTR) model at positions 1 + 2 + 3. The noise in the signal was evaluated by the likelihood mapping method implemented at the Tree-puzzle v5.3.rc16 program^59^. RDP4 v4.36 program was used to analyse recombination events within CVA24v alignments resulted from the duplex elimination for 3C and VP1 coding regions and among those strains selected and different strains of enterovirus C species (Supplementary Table S6 and S7 respectively). Potential recombinants were assumed when more than three methods implemented into the program showed significant support for recombination with a Bonferroni-corrected *P* value cut-off of 0.05^27^.

### Phylogenetic and phylodynamic analyses

In order to explore the genotypes of Cuban variants, the unique 3C and VP1 sequences were aligned and used for a heat map analysis by the R statistical package^60^. Column scaling of the heat data was performed to visualise the nucleotide identity matrix taken into account the four CVA24v genotypes as previously described^7^.

Nucleotide substitution models were estimated by jModelTest v2.1.4 program to obtain the best-fit model according to the Bayesian Information Criterion (BIC)^26^. The CVA24v phylogenetic and phylodynamic analysis were inferred by using Bayesian Inference (BI) methodology implemented into Bayesian Evolutionary Analysis Sampling Tree (BEAST) v1.8.4 program^61^. Bayesian Markov Monte Carlo Chain approach (MCMC) was used to analyse the substitution rates per site per year and the time of the most recent common ancestor (TMRCA) with 95% highest posterior density (HPD). It was also used to reconstruct the history of the viral population and spatiotemporal dynamic by using both alignments, 3C and VP1.

The clock model was selected by estimating the marginal (log) likelihood of each model using the path sampling (PS) method described by Baele et al.^62^. This simulation was carried out for models with strict or relaxed molecular clock (using an exponential uncorrelated (UCED) or a lognormal uncorrelated (UCLD) distribution) combined with the Bayesian Skyride Plot (BSP) a nonparametric coalescence model as an epidemiological model for the tree priors^63^. Bayesian MCMC analyses were run with a chain of 70 million of generations for 3C coding region and a chain of 100 million generations for VP1 coding region. Convergence parameters were identified by Tracer v1.7.1 (https://tree.bio.ed.ac.uk/software/tracer/) with the effective sample size (ESS) greater than 200 (ESS > 200). In all cases, the initial 10% of the run was used as “burn-in”.

The Maximum Clade Credibility (MCC) tree was calculated by TreeAnnotator v1.8.4 and then visualized with FigTree v1.4.4. (https://tree.bio.ed.ac.uk/software/figtree/).

Phylogeographic analysis were performed using a continuous standard type Markov chain with the Bayesian Stochastic Search Variable Selection method (BSSVS)^64^. A discrete diffusion model was used as a substitution model where the states were the countries where the virus strains were collected. The BSSVS results were visualized with the Spatial Phylogenetic Reconstruction of Evolutionary Dynamics program (SPREAD v0.9.7.1)^65^. The Bayes Factor (BF) test was performed to obtain the statistical data that adequately explained virus dispersal routes. Virus transmission events with BF > 3 were taken as significant. An additional Walktrap analyses was conducted for strains collected within countries with positive transmission routes (BF > 3) in order to detect viral transmission networks of CVA24v^32^.

### Ethics statement

All procedures performed in the study were in accordance with relevant guidelines and regulations and with the principles of the Declaration of Helsinki. The study protocol was reviewed and approved by the local ethics committee at Pedro Kourí Tropical Medicine Institute under the permit number CEI-IPK 06-17. Patient consent for the use of the samples was waived for this study in view of the fact that the research study was conducted retrospectively from samples obtained for routine diagnostics, which had been de-identified.

## Supplementary information

Supplementary Information.

## Data Availability

A list of NCBI accession number of all sequences analyzed is reported in Supplementary Table S4-S8.
