# Supplementary material for: Molecular evolution of coxsackievirus A24v in Cuba over 23-years, 1986–2009
Source: Sci Rep. 2020 Aug 13;10:13761. doi: 10.1038/s41598-020-70436-w (PMC7427094; doi:10.1038/s41598-020-70436-w)
Supplement: Supplementary file 1 — Supplementary Information. [file 41598_2020_70436_MOESM1_ESM.pdf]

Molecular evolution of coxsackievirus A24v in Cuba over 23-years, 1986-2009

Magilé C Fonseca *1, Mario Pupo-Meriño 2, Luis A García-González 3,4, Sonia Resik1, Lai Heng Hung1, Mayra Muné1, Hermis Rodríguez5, Luis Morier 6, Helene Norder 7,8**, Luis Sarmiento9**.

1. Virology Department, Center for Research, Diagnosis and Reference, Institute of Tropical Medicine "Pedro Kourí", Havana City, Cuba.

2. Departamento de Bioinformática, Centro de Matemática Computacional, Universidad de las Ciencias Informáticas (UCI), Habana, Cuba.

3. Centro de Matemática Computacional, Universidad de las Ciencias Informáticas (UCI), Habana, Cuba.

4. Departamento de Ciencias de la Computación, Centro de Investigación Científica y de Educación Superior de Ensenada, Ensenada, Baja California, México.

5. Cell Culture Laboratory, Center for Research, Diagnosis and Reference, Institute of Tropical Medicine "Pedro Kourí", Havana City, Cuba .

6. Department of Microbiology and Virology, Biology Faculty, Havana University, Havana, Cuba.

7. Department of Infectious Diseases/Virology, Institute of Biomedicine, Sahlgrenska Academy, University of Gothenburg, Gothenburg, Sweden.

8. Department of Clinical Microbiology, Region Västra Götaland, Sahlgrenska University Hospital, Gothenburg, Sweden.

9. Immunovirology Unit, Department of Clinical Sciences, Skåne University Hospital, Lund University, Malmo, Sweden.

* corresponding author: Magilé C Fonseca. Enteroviruses Diseases Laboratory, Virology Department, Pedro Kourí Institute of Tropical Medicine (IPK), Novia del Mediodía Km 61/2, La Lisa P.O. Box: 601, Marianao 13, The Havana, Cuba. Fax: (53-7) 204 60 51. Telephone: (537) 255 35 50. Web: www.ipk.sld.cu. Mail:magile@ipk.sld.cu, mcfonsecaq@gmail.com

** These authors are joint senior authors of this manuscript.

Supplementary Information.

Table S1. Number of Cuban strains used for the phylogenetic and phylodinamic analysis of 3C (507nt) and VP1 (234nt) sequences.

AHC Cuban epidemics (years)	Number of sequenced strains/specimen sources of the strains	Number of sequenced obtained/ specimen sources of the strains		Number of sequenced selected for analysis after filtering duplex/specimen sources of the strains	
		3C	VP1	3C	VP1
1986	27 CS	27 CS	27 CS	3 CS	8 CS
1987	8 CS	6 CS	5 CS	1 CS	1 CS
1992	22 CS	22 CS	22 CS	9 CS	4 CS
1993	15 CS	15 CS	13 CS	7 CS	2 CS
1997	25 CS<br>16 F<br>1 PS<br>1 NS	25 CS<br>16 F<br>1 PS<br>1 NS	20 CS<br>16 F<br>1 PS<br>1 NS	6 CS<br>8 F	7 CS<br>2 F
2003	23 CS<br>16 F	23 CS<br>16 F	23 CS<br>16 F	9 CS<br>3 F	9 CS<br>1 F
2005	5 CS	5 CS	5 CS	1 CS	1CS
**Subtotal**	**159**	**157**	**149**	**47**	**35**
2008	5 CS<br>1F	5 CS<br>1F	*ND*	1 CS	*ND*
2009	9 CS<br>1 F	9 CS<br>1 F	*ND*	5 CS<br>1F	*ND*
**Total**	**175**	**173 (123 CS, 32 F, 1PS, 1NS)**	**149 (117 CS, 30F, 1PS, 1 NS)**	**54 (42 CS, 12 F)**	**35 (32 CS, 3F)**

CS: conjunctival swabs, F: feces, PS: pharyngeal swab, NS: nasal swab,
ND: non determined, the VP1 region was not sequenced

Table S2. Nucleotide and amino acid identity between the Cuban strains and the prototype strain EH24_70_Singapore 1970 in the 3C and VP1 coding regions**.**

Cuban AHC epidemics	3C EH24_70_Singapore 1970		VP1 EH24_70_Singapore 1970	
	**% nt identity/ (N)**	**% aa identity/ (N)**	**% nt identity/ (N)**	**% aa identity/ (N)**
1986-1987	89.1-89.3/(33)	98.2-98.8/(33)	88.0-88.4/(32)	97.4/(32)
1992-1993	88.1-89.1/(37)	97.6-98.2/(37)	85.4-87.1/(35)	96.1-98.7/(35)
1997	85.9-86.7/(43)	95.8-97.0/(43)	85.4-86.7/(38)	96.1-97.4/(38)
2003/2005	86.1-86.9/(44)	95.8-96.4/(44)	83.7-85.4/(44)	93.5-94.8/(44)
2008-2009	85.7-86.1/(16)	96.4-97.0/(16)	ND	ND

N= Number of sequences
ND: non determined, the VP1 region was not sequenced

Table S3. Nucleotide identity between the 3C and VP1 coding regions of Cuban strains isolated over two consecutive epidemic years.

Cuban AHC epidemics	% nt identity between Cuban strains/(N)	
	**3C**	**VP1**
1986-1987	98.7-100/(33)	96.9-100/(32)
1992-1993	98.5-100/(37)	99.2-100/(35)
2003/2005	97.7-100/(44)	97.3-100/(44)
2008-2009	99.4-100/(16)	ND

N= Number of sequences
ND: non determined, the VP1 region was not sequenced

Table S4. Data set selected for phylogenetic and phylodinamic analysis of the 3C coding region including world-wide and Cuban CVA24v sequences. The specified genotype is based on this study.

	GenBank accession number _strain name_country_year of isolation	Genotype
1	D90457_EH24_Singapore_1_1970	GI
2	D10296_Singapore_2_1970	GI
3	D10295_Singapore_3_1970	GI
4	D10297_Hong_Kong_1_China_1971	GI
5	D10298_Singapore_1_1975	GII
6	D10299_Singapore_2_1975	GII
7	D10300_Thailand_1_1975	GII
8	D10301_Singapore_1_1985	GIII
9	D10307_Okinawa_1_Japan_1985	GIII
10	D13270_L062_85_Taiwan_1985	GIII
11	D10267_Kanagawa_1_Japan_1986	GIII
12	D13273_V150_Taiwan_1986	GIII
13	D13272_V116_86_Taiwan_1986	GIII
14	FJ042688_Taiwan_1986	GIII
15	D10311_Okinawa_1_Japan_1986	GIII
16	D10303_Henan_1_China_1986	GIII
17	D10302_Shanghai_1_China_1986	GIII
18	D10315_Islamabad_Pakistan_1986	GIII
19	KC128616_CUBA_1986	GIII
20	KC128631_CUBA_1986	GIII
21	KC128638_CUBA_1986	GIII
22	KC128643_CUBA_1987	GIII
23	D10312_Ghana_1_1987	GIII
24	D10313_Ghana_2_1987	GIII
25	D10314_Ghana_3_1987	GIII
26	EF015037_Jamaica_1987	GIII
27	EF015038_Brazil_1987	GIII
28	GU983207_Brazil_1987	GIII
29	D10324_Kaohsiung_2_Taiwan_1988	GIII
30	D10322_Singapore_1_1988	GIII
31	D13278_590_Taiwan_1988	GIII
32	D13285_865_89_Taiwan_1989	GIII
33	D10277_Mobara_1_Japan_1989	GIII
34	AB008476_062_Taiwan_1990	GIII
35	KC205691_CUBA_1992	GIII
36	KC205693_CUBA_1992	GIII
37	KC205692_CUBA_1992	GIII
38	KC205695_CUBA_1992	GIII
39	KC205698_CUBA_1992	GIII
40	KC205702_CUBA_1992	GIII
41	KC205710_CUBA_1992	GIII
42	KC205711_CUBA_1992	GIII
43	KC205712_CUBA_1992	GIII
44	KC205713_CUBA_1993	GIII
45	KC205714_CUBA_1993	GIII

	GenBank accession number _strain name_country_year of isolation	Genotype
46	KC205715_CUBA_1993	GIII
47	KC205717_CUBA_1993	GIII
48	KC205720_CUBA_1993	GIII
49	KC205722_CUBA_1993	GIII
50	KC205726_CUBA_1993	GIII
51	AB008485_46_Thailand_1993	GIII
52	AB008484_26_Thailand_1993	GIII
53	EF015039_Dominican_Republic_1993	GIII
54	AB008481_072_Taiwan_1994	GIII
55	AB008480_066_Taiwan_1994	GIII
56	AB008479_063_Taiwan_1994	GIII
57	AB008477_042_Taiwan_1994	GIII
58	EF015040_USA_1998	GIV**
59	KC286917_CUBA_1997	GIV**
60	KC286918_CUBA_1997	GIV**
61	KC286919_CUBA_1997	GIV**
62	KC286924_CUBA_1997	GIV**
63	KC286927_CUBA_1997	GIV**
64	KC286937_CUBA_1997	GIV**
65	KC286938_CUBA_1997	GIV**
66	KC286940_CUBA_1997	GIV**
67	KC286942_CUBA_1997	GIV**
68	KC286945_CUBA_1997	GIV**
69	KC286946_CUBA_1997	GIV**
70	KC286947_CUBA_1997	GIV**
71	KC286948_CUBA_1997	GIV**
72	KC286949_CUBA_1997	GIV**
73	DQ472129_Taiwan_2000	GIV
74	DQ472138_Taiwan_2000	GIV
75	DQ472136_Taiwan_2001	GIV
76	AB473409_Taiwan_2001	GIV
77	DQ472134_Taiwan_2002	GIV
78	DQ472137_Taiwan_2002	GIV
79	AY216777_South_Korea_2002	GIV
80	AY216782_South_Korea_2002	GIV
81	AY216789_South_Korea_2002	GIV
82	AY216790_South_Korea_2002	GIV
83	AY876913_Hangzhou13_02_China_2002	GIV
84	AY942640_Mumbai4_03_India_2003	GIV
85	AY942641_Pune6-03_India_2003	GIV
86	AY876182_French_Guiana_2003	GIV
87	AY876183_French_Guiana_2003	GIV
88	AY876186_Guadeloupe_2003	GIV
89	AY876187_Guadeloupe_2003	GIV
90	AY876191_Guadeloupe_2003	GIV
91	AY876192_Guadeloupe_2003	GIV
92	GU983208_Brazil_2003	GIV
93	GU983212_Brazil_2003	GIV

	GenBank accession number _strain name_country_year of isolation	Genotype
94	GU983221_Brazil_2003	GIV
95	GU983230_Brazil_2004	GIV
96	GU983232_Brazil_2004	GIV
97	KC286994_CUBA_2003	GIV
98	KC286998_CUBA_2003	GIV
99	KC287000_CUBA_2003	GIV
100	KC287005_CUBA_2003	GIV
101	KC287010_CUBA_2003	GIV
102	KC287011_CUBA_2003	GIV
103	KC287012_CUBA_2003	GIV
104	KC287013_CUBA_2003	GIV
105	KC287014_CUBA_2003	GIV
106	KC287016_CUBA_2003	GIV
107	KC287022_CUBA_2003	GIV
108	KC287025_CUBA_2003	GIV
109	KC287037_CUBA_2005	GIV
110	DQ370152_0382_04_DR_Congo_2004	GIV
111	DQ370147_0370_04_DR_Congo_2004	GIV
112	DQ370146_0365_04_DR_Congo_2004	GIV
113	DQ370162_01531_04_Morocco_2004	GIV
114	DQ370160_01529_04_Morocco_2004	GIV
115	DQ370159_01525-04_Morocco_2004	GIV
116	DQ443002_Singapore_2005	GIV
117	DQ443001_Singapore_2005	GIV
118	GU983236_Brazil_2005	GIV
119	GU983238_Brazil_2005	GIV
120	FJ200506_Taiwan_2006	GIV
121	FJ232037_Taiwan_2006	GIV
122	FJ200514_Taiwan_2007	GIV
123	FJ042700_Taiwan_2007	GIV
124	FJ042701_Taiwan_2007	GIV
125	GU477563_India_2007	GIV
126	GU477564_India_2007	GIV
127	GU477568_India_2007	GIV
128	HM156623_CUBA_2008	GIV
129	HM156613_CUBA_2009	GIV
130	HM156616_CUBA_2009	GIV
131	HM156617_CUBA_2009	GIV
132	HM156618_CUBA_2009	GIV
133	HM156621_CUBA_2009	GIV
134	HM156622_CUBA_2009	GIV
135	GU983241_Brazil_2009	GIV
136	GU983242_Brazil_2009	GIV
137	GU983243_Brazil_2009	GIV

GIV** Strains classified within GIII genotype described by Chu et. al, 2009 (1)

Table S5. Data set selected for phylogenetic and phylodinamic analysis of the VP1 coding region including world-wide and Cuban CVA24v sequences. The specified genotype is based on this study.

	GenBank accession number _strain name_country_year of isolation	Genotype
1	D90457_EH24_70_Singapore_1970	GI
2	AB473429_L001_Taiwan_1985	GIII
3	AB473430_V150_Taiwan_1986	GIII
4	KC184859_CUBA_1986	GIII
5	KC184869_CUBA_1986	GIII
6	KC184872_CUBA_1986	GIII
7	KC184873_CUBA_1986	GIII
8	KC184875_CUBA_1986	GIII
9	KC184877_CUBA_1986	GIII
10	KC184879_CUBA_1986	GIII
11	KC184881_CUBA_1986	GIII
12	KC184886_CUBA_1987	GIII
13	EF015038_Brazil_1987	GIII
14	GU983190_PA_1_Brazil_1987	GIII
15	EF015037_Jamaica_1987	GIII
16	AB473432_865_Taiwan_1989	GIII
17	AB473431_804_Taiwan_1989	GIII
18	EF015039_Dominican_Republic_1993	GIII**
19	KC205656_CUBA_1991	GIII
20	KC205664_CUBA_1992	GIII
21	KC205675_CUBA_1992	GIII
22	KC205676_CUBA_1992	GIII
23	KC205682_CUBA_1993	GIII
24	KC205690_CUBA_1993	GIII
25	*95_PHL_1996_Philippines_1996	GIV**
26	*107_PHL_1996_Philippines_1996	GIV**
27	*174_PHL_1996_Philippines_1996	GIV**
28	*111_PHL_1997_Philippines_1997	GIV**
29	KC286959_CUBA_1997	GIV**
30	KC286960_CUBA_1997	GIV**
31	KC286963_CUBA_1997	GIV**
32	KC286966_CUBA_1997	GIV**
33	KC286972_CUBA_1997	GIV**
34	KC286977_CUBA_1997	GIV**
35	KC286981_CUBA_1997	GIV**
36	KC286983_CUBA_1997	GIV**
37	KC286988_CUBA_1997	GIV**
38	EF015040_Texas_USA_1998	GIV**
39	AY208105_98_30257_38_99_France_1998	GIV**
40	AB473433_Taiwan_2000	GIV
41	*14_PHL_2000_Philippines_2000	GIV
42	*15_PHL_2000_Philippines_2000	GIV
43	*28_PHL_2000_Philippines_2000	GIV
44	*30_PHL_2000_Philippines_2000	GIV
45	*31_PHL_2000_Philippines_2000	GIV
46	*159_PHL_2000_Philippines_2000	GIV
47	AB473434_Taiwan_2001	GIV

	GenBank accession number _strain name_country_year of isolation	Genotype
48	AB473435_Taiwan_2001	GIV
49	*63_PHL_2002_Philippines_2002	GIV
50	*172_PHL_2002_Philippines_2002	GIV
51	DQ434857_South_Korea_2002	GIV
52	DQ434858_South_Korea_2002	GIV
53	DQ434861_South_Korea_2003	GIV
54	DQ434866_South_Korea_2003	GIV
55	GQ329725_China_2003	GIV
56	AY876169_French_Guiana_2003	GIV
57	AY876174_Guadeloupe_2003	GIV
58	AY876175_Guadeloupe_2003	GIV
59	AY876179_Guadeloupe_2003	GIV
60	GU983184_Brazil_2003	GIV
61	GU983186_Brazil_2003	GIV
62	GU983187_Brazil_2003	GIV
63	GU983193_Brazil_2004	GIV
64	GU983196_Brazil_2004	GIV
65	KC287038_CUBA_2003	GIV
66	KC287040_CUBA_2003	GIV
67	KC287048_CUBA_2003	GIV
68	KC287049_CUBA_2003	GIV
69	KC287053_CUBA_2003	GIV
70	KC287054_CUBA_2003	GIV
71	KC287055_CUBA_2003	GIV
72	KC287056_CUBA_2003	GIV
73	KC287058_CUBA_2003	GIV
74	KC287064_CUBA_2003	GIV
75	KC287080_CUBA_2005	GIV
76	AB365074_Pakistan_2004	GIV
77	AB365075_Pakistan_2004	GIV
78	AB365076_Pakistan_2004	GIV
79	EU162074_Spain_2004	GIV
80	EU162077_Spain_2004	GIV
81	GU983199_Brazil_2005	GIV
82	GU983201_Brazil_2005	GIV
83	GU983203_Brazil_2005	GIV
84	DQ443001_Singapore_2005	GIV
85	DQ443002_Singapore_2005	GIV
86	DQ901736_Singapore_2005	GIV
87	FJ232034_Taiwan_2006	GIV
88	AB473436_Taiwan_2006	GIV
89	EF176672_South_Korea_2006	GIV
90	EF176676_South_Korea_2006	GIV
91	FJ868371_Australia_2006	GIV
92	GQ229398_China_2007	GIV
93	GU477573_India_2007	GIV
94	GU477579_India_2007	GIV
95	GU477580_India_2007	GIV
96	GU477582_India_2007	GIV
97	EU391662_China_2007	GIV
98	GQ429279_China_2007	GIV
99	AB473439_Taiwan_2007	GIV
100	AB473440_Taiwan_2007	GIV

	GenBank accession number _strain name_country_year of isolation	Genotype
101	AB473441_Taiwan_2007	GIV
102	JX538080_14662_Bangladesh_2007	GIV
103	JX538079_14661_Bangladesh_2007	GIV
104	GQ429280_China_2008	GIV
105	GQ429287_China_2008	GIV
106	GQ429288_China_2008	GIV
107	JQ744321_30_Ma_223_PHL_Philippines_2008	GIV
108	JQ744322_33_LP_172_PHL_Philippines_2008	GIV
109	JQ744323_30_Ma_221_PHL_Philippines_2008	GIV
110	JQ744324_20_Pa_235_PHL_Philippines_2008	GIV
111	JQ744317_38_Me_271_PHL_Philippines_2008	GIV
112	*128_PHL_2008_Philippines_2008	GIV
113	JX538127_709011_Bangladesh_2008	GIV
114	JX538128_709012_Bangladesh_2008	GIV
115	JX538132_709041_Bangladesh_2008	GIV
116	JX538205_14793_Bangladesh_2008	GIV
117	JX538207_14795_Bangladesh_2008	GIV
118	JX538211_14806_Bangladesh_2008	GIV
119	JX538213_14808_Bangladesh_2008	GIV
120	JX538214_14809_Bangladesh_2008	GIV
121	JX538216_14815_Bangladesh_2008	GIV
122	JX538220_14820_Bangladesh_2008	GIV
123	JX417879_24v_g08_005_Gabon_2008	GIV
124	KF667358_INDNIV1034661LV463_India_2009	GIV
125	KF667359_INDNIV1036731LV476_India_2009	GIV
126	KF667360_INDNIV1044161LV530_India_2009	GIV
127	KF667361_INDNIV1040633LV639_India_2009	GIV
128	GU983204_Brazil_2009	GIV
129	GU983205_Brazil_2009	GIV
130	GU983206_Brazil_2009	GIV

* Sequences provided by professor Lea Necitas G Apostol, RMT, MPH, PhD from the Research Institute for Tropical Medicine-Department of Health and Dr. Oshitani from Tohoku University Graduate School of Medicine of Philippines (2, 3) .

GIV** Strains classified within GIII genotype described by Chu et. al.2009 (1).

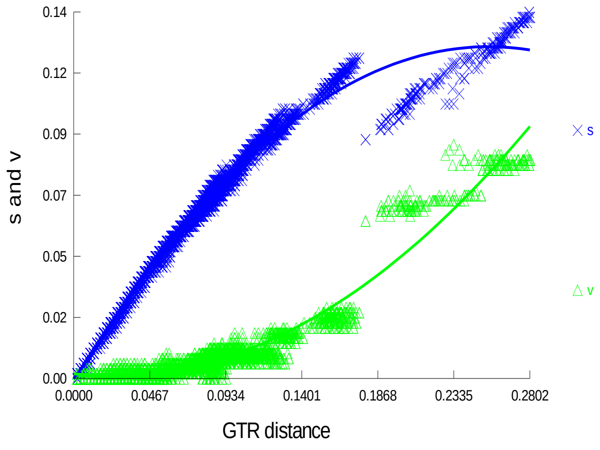

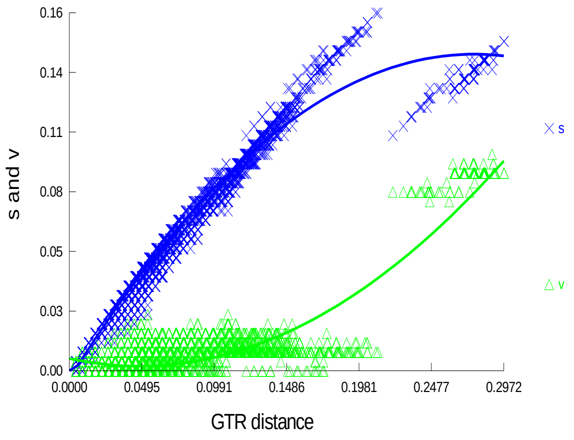


```
Test of substitution saturation (Xia et al. 2003; Xia and Lemey 2009)

Analysis performed on all sites.

Testing whether the observed Iss is significantly lower than Iss.c.
  IssSym is Iss.c assuming a symmetrical topology.
  IssAsym is Iss.c assuming an asymmetrical topology.

NumOTU      Iss  Iss.cSym       T    DF        P Iss.cAsym       T    DF        P
================================================================================
     4    0.105     0.796  41.932   377   0.0000     0.762  39.861   377   0.0000
     8    0.107     0.752  36.959   377   0.0000     0.641  30.567   377   0.0000
    16    0.109     0.721  35.207   377   0.0000     0.512  23.191   377   0.0000
    32    0.111     0.703  34.183   377   0.0000     0.378  15.404   377   0.0000
Note: two-tailed tests are used.

================================================================================
Interpretation of results:
              Significant Difference
              ----------------------
              Yes                 No
---------------------------------------------------------
Iss < Iss.c   Little              Substantial
              saturation          saturation
---------------------------------------------------------
Iss > Iss.c   Useless             Very poor
              sequences           for phylogenetics
----------------------------------------------------------------
```

```
Test of substitution saturation (Xia et al. 2003; Xia and Lemey 2009)

Analysis performed on all sites.

Testing whether the observed Iss is significantly lower than Iss.c.
  IssSym is Iss.c assuming a symmetrical topology.
  IssAsym is Iss.c assuming an asymmetrical topology.

NumOTU      Iss  Iss.cSym       T    DF        P Iss.cAsym       T    DF        P
================================================================================
     4    0.136     0.777  21.811   133   0.0000     0.761  21.264   133   0.0000
     8    0.132     0.732  20.412   133   0.0000     0.630  16.926   133   0.0000
    16    0.127     0.656  18.729   133   0.0000     0.460  11.769   133   0.0000
    32    0.134     0.684  19.652   133   0.0000     0.362   8.135   133   0.0000
Note: two-tailed tests are used.

================================================================================
Interpretation of results:
              Significant Difference
              ----------------------
              Yes                 No
---------------------------------------------------------
Iss < Iss.c   Little              Substantial
              saturation          saturation
---------------------------------------------------------
Iss > Iss.c   Useless             Very poor
              sequences           for phylogenetics
----------------------------------------------------------------
```

3C coding region **VP1 coding region**

Figure S1. Transitions and transversions against a corrected genetic distance plotting using DAMBE analysis (top) and Xia test results (bottom) for 3C (left) and VP1 (right) coding regions.

3C coding region

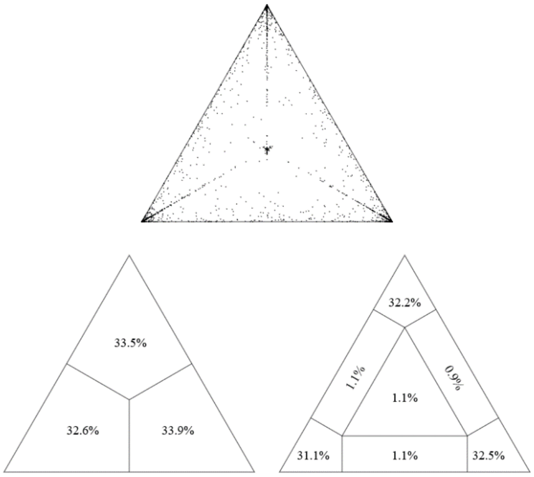

VP1 coding region

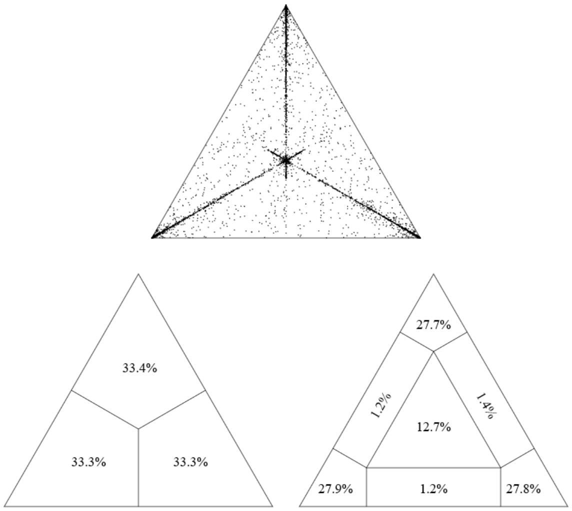

Figure S2. TreePuzzle analysis for 3C (top) and VP1 coding regions (bottom).

Table S6. *Enterovirus C* strains selected for the recombination test on 3C (507nt) coding region.

**GenBank accession number _enterovirus serotype_strain name**
AF499635_Human_coxsackievirus_A1_strain_Tompkins
AF499636_Human_coxsackievirus_A11_strain_Belgium-1
DQ995644_Human_coxsackievirus_A13_strain_USA/Ca98-10615
AF499638_Human_coxsackievirus_A15_strain_G9
AF499639_Human_coxsackievirus_A17_strain_G12
AF499640_Human_coxsackievirus_A18_strain_G13
AF499641_Human_coxsackievirus_A19_strain_8663
EF015014_Human_coxsackievirus_A20_strain_BAN01-10618
EF015016_Human_coxsackievirus_A20_strain_BAN99-10635
EF015028_Human_coxsackievirus_A21_strain_GUT88-10687
EF015030_Human_coxsackievirus_A21_strain_BAN00-10552
EF015024_Human_coxsackievirus_A21_strain_USA-Ga95-10622
AF499643_Human_coxsackievirus_A22_strain_Chulman
EF026081_Human_coxsackievirus_A24_Joseph_South_Africa_1952
AY082689_Human_poliovirus_1_strain_Mahoney
AY082680_Human_poliovirus_2_strain_Lansing
EF015009_Human_enterovirus_99_strain_BAN01-10582
EF555645_Human_enterovirus_102
AB686524_Human_enterovirus_C104_ strain_AK11
EU840733_Human_enterovirus_104_strain_CL-12310945
GQ865517_Human_enterovirus_109_isolate_NICA08-4327

Table S7. *Enterovirus C* strains selected for the recombination test on VP1 (234nt) coding region.

**GenBank accession number _enterovirus serotype_strain name**
AF499635_Human_coxsackievirus_A1_strain_Tompkins
AF499636_Human_coxsackievirus_A11_strain_Belgium-1
DQ995644_Human_coxsackievirus_A13_strain_USA/Ca98-10615
AF499638_Human_coxsackievirus_A15_strain_G9
AF499639_Human_coxsackievirus_A17_strain_G12
AF499640_Human_coxsackievirus_A18_strain_G13
AF499641_Human_coxsackievirus_A19_strain_8663
AF499643_Human_coxsackievirus_A22_strain_Chulman
EF015016_Human_coxsackievirus_A20_strain_BAN99-10635
EF015014_Human_coxsackievirus_A20_strain_BAN01-10618
EF015030_Human_coxsackievirus_A21_strain_BAN00-10552
EF015024_Human_coxsackievirus_A21_strain_USA-Ga95-10622
EF015028_Human_coxsackievirus_A21_strain_GUT88-10687
AF081311_Coxsackievirus_A24_strain_Joseph
KF129002_Coxsackievirus_A24_isolate_FIN06-EV06-32A-30807
AY082689_Human_poliovirus_1_strain_Mahoney
AY082680_Human_poliovirus_2_strain_Lansing
V01132_Poliovirus_type_3_Leon
HQ415759_Human_enterovirus_96_strain_09228C1
EF364404_Human_enterovirus_96_isolate_SVK03-24
EF015009_Human_enterovirus_99_strain_BAN01-10582
KF129046_Enterovirus_C99_isolate_RUS00-13831
EF555645_Human_enterovirus_102
EU840733_Human_enterovirus_104_strain_CL-12310945
AB686524_Human_enterovirus_C104_ strain_AK11
GQ865517_Human_enterovirus_109_isolate_NICA08-4327

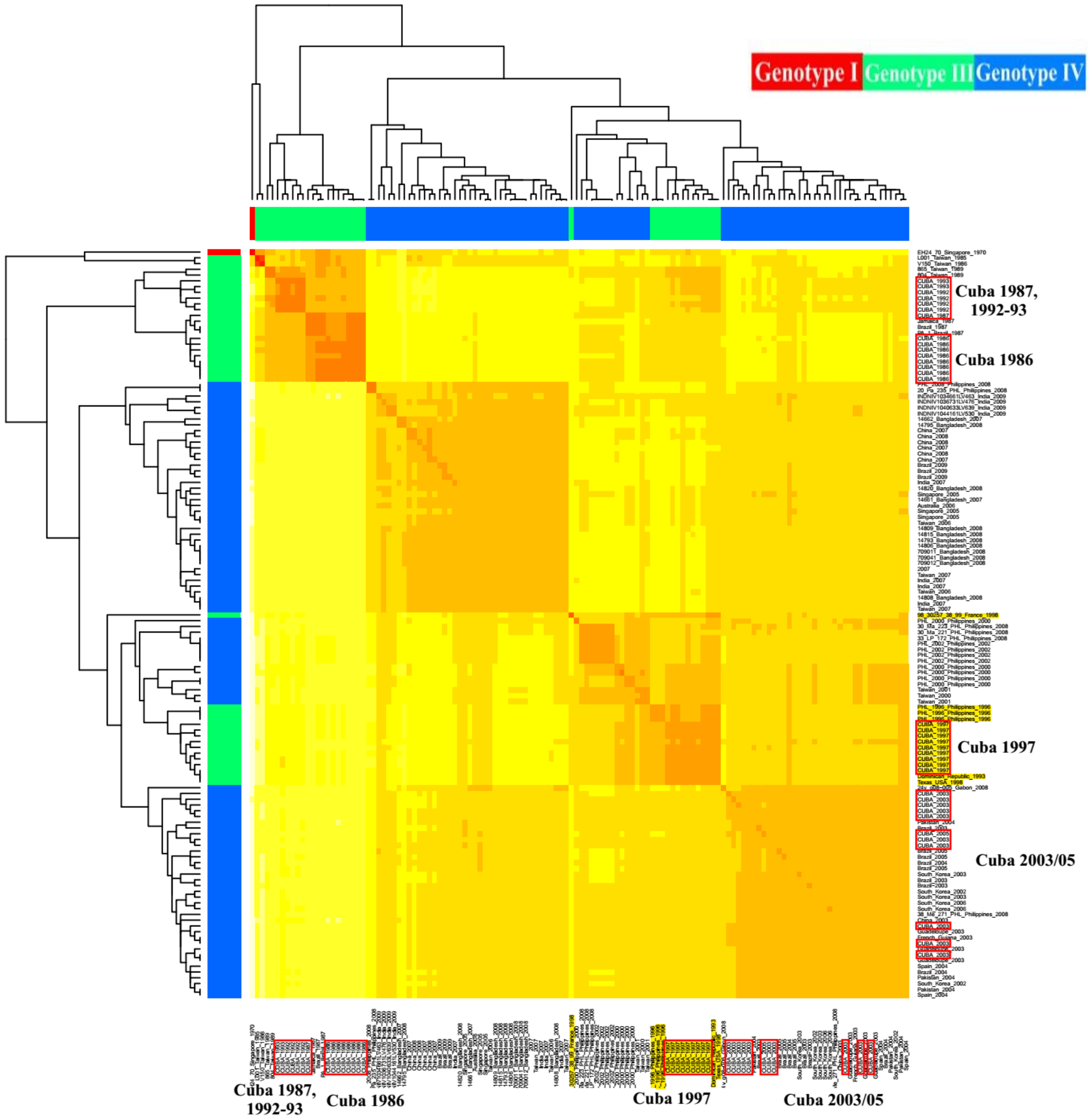

Figure S3. Heat map from nucleotide identity matrix of the CVA24v alignment of VP1 genomic region. Sequences were classified by GI-IV Genotypes described by Chu et.al. (1). Genotypes are indicated by the colors legend on the top side. French sequence (1998), Cuban sequences (1997), Philippines sequences (1996), USA sequence (1998) and Dominique Republic sequence (1993) are highlighted in yellow. Cuban sequences from the five AHC epidemics analyzed are highlighted in red rectangles.

Table S8. *Enterovirus C* strains (complete genome) selected to the recombination test for the 1993 Dominican strain (EF015039 strain DOR93-10630).

**GenBank accession number _enterovirus serotype_strain name**
D90457_ Human_ coxsackievirus _A24_variant_strain_ EH24_70_Singapore_1970
JF742579_Human _ coxsackievirus _A24_variant_strain_ China_GD332_2007
JF742578_Human _ coxsackievirus _A24_variant_strain_ China_GD391-2007
JF742577_Human _ coxsackievirus _A24_variant_strain_ China_GD01_2010
JF742576_Human _ coxsackievirus _A24_variant_strain_ China_GD46_2010
JN228097_Human _ coxsackievirus _A24_variant_ South Korea_ 2004
DQ443002_Human _ coxsackievirus _A24_variant_strain_ Singapore_2005
DQ443001_Human _ coxsackievirus _A24_variant_strain_ Singapore_2005
EF026081_Human _ coxsackievirus _A24_variant_strain_ Joseph_South_Africa_1952
KF667361_Human _ coxsackievirus _A24_variant_strain_ INDNIV1040633LV639_India_2009
KF667360_Human _ coxsackievirus _A24_variant_strain_ INDNIV1044161LV530_India_2009
KF667359_Human _ coxsackievirus _A24_variant_strain_ INDNIV1036731LV476_India_2009
KF667358_Human _ coxsackievirus _A24_variant_strain_ INDNIV1034661LV463_India_2009
MF419263_Human _ coxsackievirus _A24_variant_strain_GUF_2017_B0519081_French Guiana_2017
KF725085_Human _ coxsackievirus _A24_variant_strain_ isolate 110390_ Malaysia_2002
EF01503_Human 7_ coxsackievirus _A24_variant_strain_JAM87-10628
EF015039_Human _ coxsackievirus _A24_variant_strain__strain_DOR93-10630
EF015038_Human _ coxsackievirus _A24_variant_strain__strain_BRA87-10629
AF081311_Human _coxsackievirus_A24_strain_Joseph
KF129002_Human _coxsackievirus_A24_isolate_FIN06-EV06-32A-30807
AF499636_Human_coxsackievirus_A11_strain_Belgium-1
DQ995644_Human_coxsackievirus_A13_strain_USA/Ca98-10615
AF499638_Human_coxsackievirus_A15_strain_G9
AF499639_Human_coxsackievirus_A17_strain_G12
AF499640_Human_coxsackievirus_A18_strain_G13
AF499641_Human_coxsackievirus_A19_strain_8663
AF499643_Human_coxsackievirus_A22_strain_Chulman
EF015014_Human_coxsackievirus_A20_strain_BAN01-10618
EF015016_Human_coxsackievirus_A20_strain_BAN99-10635
EF015028_Human_coxsackievirus_A21_strain_GUT88-10687
EF015030_Human_coxsackievirus_A21_strain_BAN00-10552
EF015024_Human_coxsackievirus_A21_strain_USA-Ga95-10622
HQ415759_Human_enterovirus_96_strain_09228C1
EF364404_Human_enterovirus_96_isolate_SVK03-24
EF015009_Human_enterovirus_99_strain_BAN01-10582
KF129046_Human_enterovirus_99_isolate_RUS00-13831
EF555645_Human_enterovirus_102
EU840733_Human_enterovirus_104_strain_CL-12310945
AB686524_Human_enterovirus_C104_genomic_RNA_strain:_AK11
GQ865517_Human_enterovirus_109_isolate_NICA08-4327
AY082689_Human_poliovirus_1_strain_Mahoney
AY082680_Human_poliovirus_2_strain_Lansing
V01132_Poliovirus_type_3_strain_Leo

Table S9. RDP4 analysis information regarding the two recombination breakpoints that were found on the 1993 Dominican strain (EF015039 strain DOR93-10630).

Breakpoint Positions									Detection Methods (p-values)								
In Alignment		In Recombinant Sequence		Relative to D90457													
Begin	End	Begin	End	Begin	End	Recombinant Sequence	Minor Parental Sequence	Major Parental Sequence	RDP	GENECONV	Bootscan	Maxchi	Chimaera	SiSscan	PhylPro	LARD	3Seq
8	3899	5	3890 ▲	5	3890	EF015039	EF026081	JN228097	NS	0.0096	1.69E-02	1.85E-13	0.9776	NS	NS	NS	NS
9*	805	6*	796 ■	6*	796	EF015039	JN228097	EF01503	1.08E-02	NS	1.45E-02	4.51E-05	5.8E-02	NS	NS	NS	NS

* = The actual breakpoint position is undetermined (it was most likely overprinted by a subsequent recombination event).
Minor Parent = Parent contributing the smaller fraction of sequence.
Major Parent = Parent contributing the larger fraction of sequence.
NS = No significant P-value was recorded for this recombination event using this method.
EF015039= strain DOR93-10630
EF026081= Human_coxsackievirus_A24_Joseph_South_Africa_1952
JN228097= Human _coxsackievirus _A24_variant_South Korea_ 2004
EF01503= JAM87-10628
▲ Breakpoint within 2B nonstructural protein
■ Breakpoint within VP4 structural protein

3C coding region

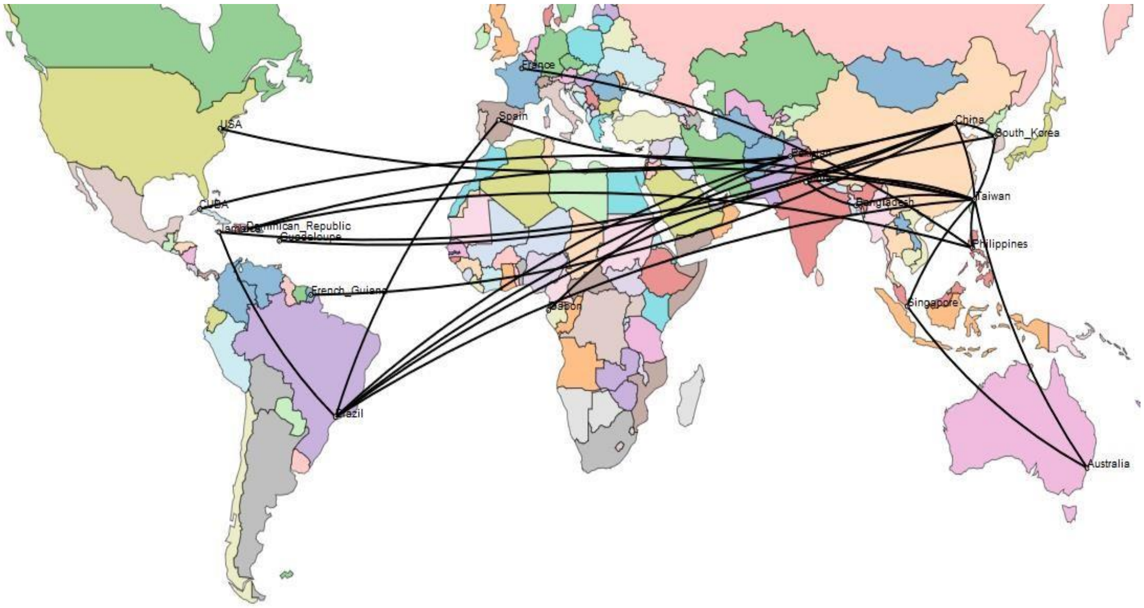

VP1 coding region

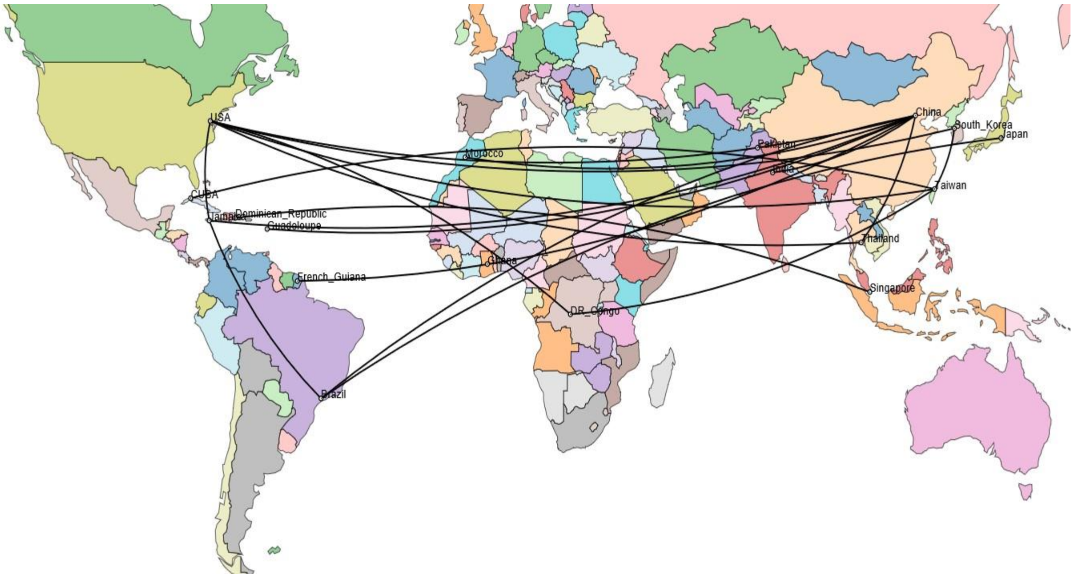

Figure S4. Spatiotemporal dynamics inferred for CVA24v based on 3C (top) and VP1 (bottom) coding region. The diffusion process was visualized using SPREAD software.

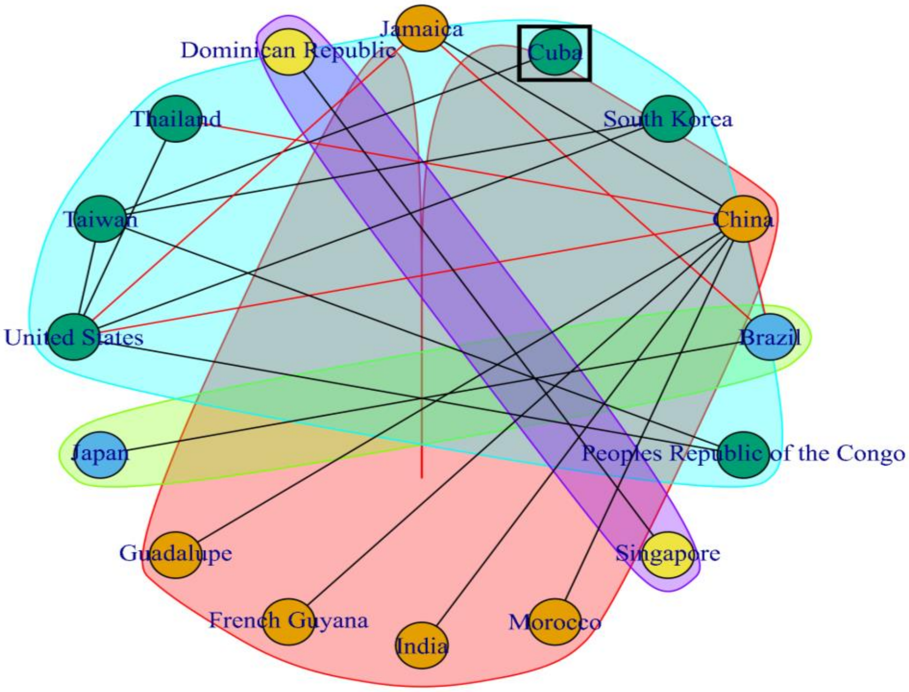

3C coding region

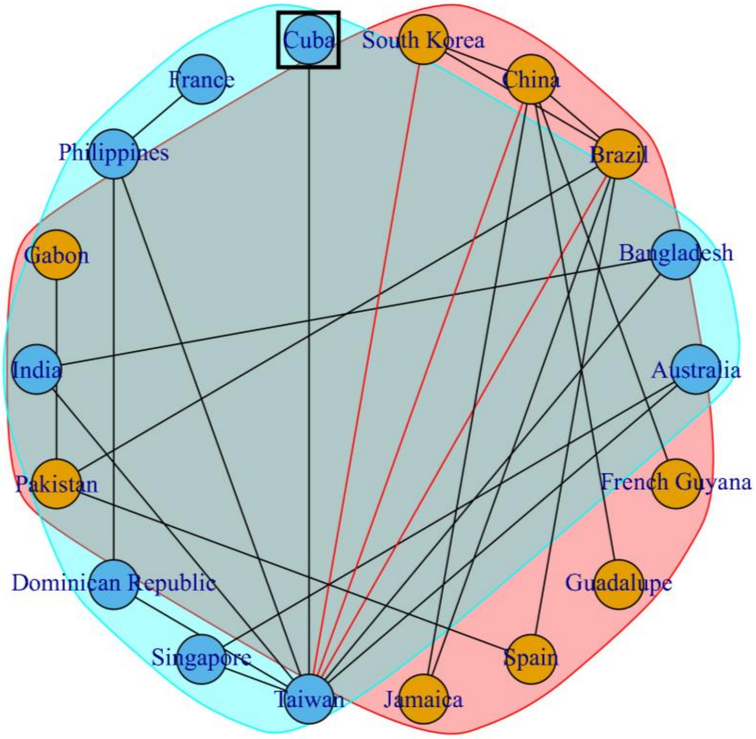

VP1 coding region

Figure S5. Walktrap community for 3C coding region (top) and VP1 coding region (bottom). Analysis is based on the viral transmission routes with BF>3 obtained from the phylogeographic analysis constructed by Bayesian method with BEAST. Cuban position in the network is highlighted in black square.

References

1. Chu, P.Y. *et al*. Molecular epidemiology of coxsackie A type 24 variant in Taiwan, 2000–2007. *J Clin Virol*. **45**, 285–291, doi:10.1016/j.jcv (2009).

2. Apostol, L.N.G., *et al*. Genetic diversity and molecular characterization of enteroviruses from sewage-polluted urban and rural rivers in the Philippines. *Virus Genes*. **45,** 207–217, https://doi.org/10.1007/s11262-012-0776-z (2012).

3. Apostol, L.N.G., *et al*. Detection of non-Polio enteroviruses from 17 years of virological surveillance of acute flaccid paralysis in the Philippines. *J Med Virol*. **84**, 624-31, doi: 10.1002/jmv.23242 (2012).
